# Genetic Algorithm (GA)-Based Inclinometer Layout Optimization

**DOI:** 10.3390/s150409136

**Published:** 2015-04-17

**Authors:** Weijie Liang, Ping Zhang, Xianping Chen, Miao Cai, Daoguo Yang

**Affiliations:** 1 The Faculty of Mechanical & Electrical Engineering, Guilin University of Electronic Technology, Guilin 541004, China; E-Mails: excelsior619@sina.com (W.L.); caimiao105@163.com (M.C.); daoguo_yang@vip.163.com (D.Y.); 2 School of Computer Science and Engineering, The University of New South Wales, 2052 Sydney, Australia; 3 Institute of Microelectronics, Tsinghua University, 100084 Beijing, China; 4 Guangxi Experiment Center of Information Science, No.1 Jinji Road, Guilin 541004, China

**Keywords:** airflow inclinometer, sensitivity study, GA, thermal layout

## Abstract

This paper presents numerical simulation results of an airflow inclinometer with sensitivity studies and thermal optimization of the printed circuit board (PCB) layout for an airflow inclinometer based on a genetic algorithm (GA). Due to the working principle of the gas sensor, the changes of the ambient temperature may cause dramatic voltage drifts of sensors. Therefore, eliminating the influence of the external environment for the airflow is essential for the performance and reliability of an airflow inclinometer. In this paper, the mechanism of an airflow inclinometer and the influence of different ambient temperatures on the sensitivity of the inclinometer will be examined by the ANSYS-FLOTRAN CFD program. The results show that with changes of the ambient temperature on the sensing element, the sensitivity of the airflow inclinometer is inversely proportional to the ambient temperature and decreases when the ambient temperature increases. GA is used to optimize the PCB thermal layout of the inclinometer. The finite-element simulation method (ANSYS) is introduced to simulate and verify the results of our optimal thermal layout, and the results indicate that the optimal PCB layout greatly improves (by more than 50%) the sensitivity of the inclinometer. The study may be useful in the design of PCB layouts that are related to sensitivity improvement of gas sensors.

## 1. Introduction

In the field of inertial technology, the mechanism of angle and acceleration measurements is mostly based on the principles of solid or liquid pendulums [1,2]. The sensing elements within a solid pendulum and liquid pendulum are relatively heavier due to the fact the sensing elements are solids or liquids. They will often get stuck due to their large inertia under strong shock and vibration conditions. Recently a new concept of a thermal accelerometer with no proof mass has been reported. The earliest principle of airflow inertial devices was studied by the Dao *et al.* [3,4,5,6,7], and micro-machined airflow inclinometers have been developed as a new type of inertial sensors based on the gas pendulum theory [8,9,10].

**Figure 1:**
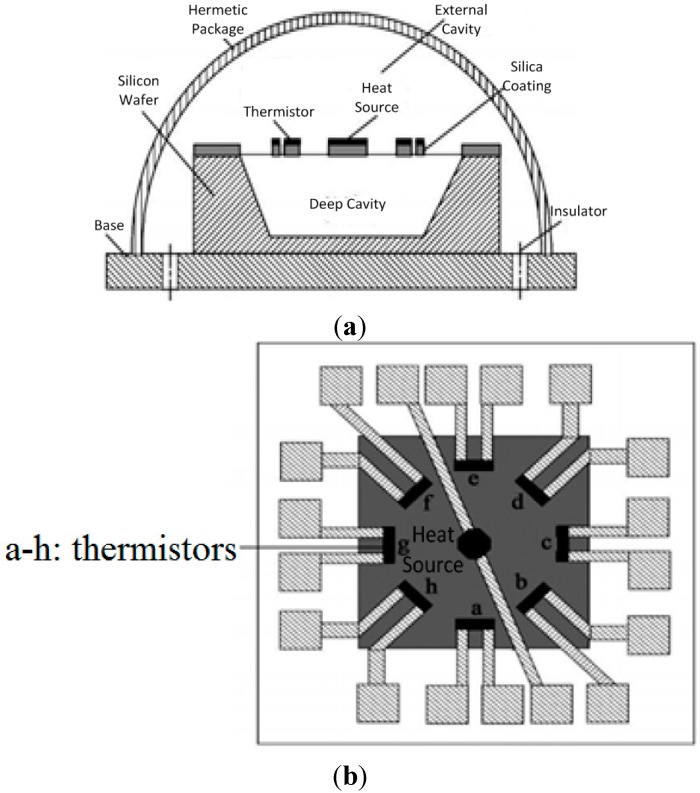

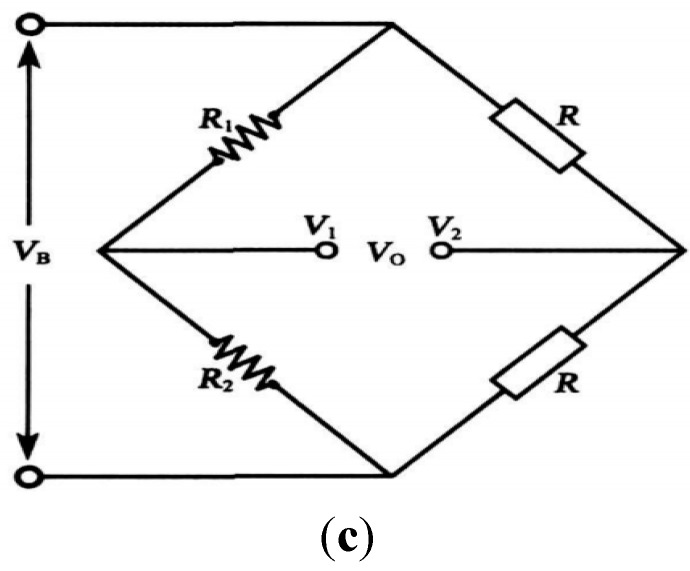
Schematic diagram of the structure of the sensing element for a micro-machined airflow inclinometer. (**a**) Schematic diagram of the sensing element; (**b**) Platform diagram of the sensing element; (**c**) Schematic of the detection bridge circuit within the sensing element.

The PCBs of airflow inclinometers consist of different components like operational amplifiers, power sources and sensing elements, *etc.* The sensing element as the sensing part of the airflow inclinometer will create an output signal related to the angle of the sensor by measuring the internal temperature difference within the sensing element. Changes in the ambient environment around the airflow inclinometer could affect the stability of the original signal detected by the sensing element, which may cause voltage drifts of the final output signal associated with tilt angles. Figure 1a shows the structure of the sensing element for a micro-machined airflow inclinometer.

Micro-machining is used to etch a deep cavity on a silicon wafer. After that, the thermistors and heat source are fixed on top of the deep cavity. A hermetic chamber is formed by the packaging process. As we can see from Figure 1b, the thermistors on the silicon wafer surround the heat source. The heat source is heated by a constant current, forming a stable temperature field within the hermetic chamber. Two thermistors (detectors) which are placed in parallel form the two arms of the bridge circuit, respectively, as shown in Figure 1c. Two identical precision linear resistors *R*_1_ and *R*_2_ constitute the two reference arms of the bridge circuit. When the inclinometer moves around the horizontal axis in some arbitrary orientation (azimuthal angle A = 0°~360°), the relative thermistor value will change according due to the changes of the temperature field they detect, and the bridge circuit will generate a voltage signal related to the tilt angle of the inclinometer. According to the mechanism of the micro-machined airflow inclinometer, any change of the environmental temperature will generate a drift current in the detection bridge circuit and a temperature drift affecting the sensor’s sensitivity. So far most studies have focused on the effect of types of working fluid media, sensor position, acceleration, shape and structure of the package and heater power on the sensitivity of gas-flowing sensors [11,12,13,14], but there are few reports in the past studies referring to the effects of ambient temperature on the gas-flowing sensor sensitivity. Different distributions of components will result in different thermal distributions on the PCB for an airflow inclinometer, which could change the surrounding temperature of the sensing element accordingly. The effect of the surrounding temperature on the sensing element is a critical factor for the sensitivity of the airflow inclinometer in actual application, so that it is necessary for the PCB layout of the airflow inclinometer to be optimized and redesigned in order to improve the sensitivity of the device. The problem of optimizing the PCB thermal layout of electronic components is a combinatorial optimization problem, which is similar to the traveling salesman problem (TSP) [15]. The field of combinatorial optimization problems is a classic application for the GA, for some non-linear and multi-objective function optimization problems and complex combinatorial optimization problems are difficult to solve by other optimization methods, but the GA can easily get better results. Using GA to solve TSP is a currently a hot research topic [16,17,18,19]. GA has been proved to be a good application for device placement, PCB assembly planning, layout problems, thermal optimization, *etc.* [20,21,22,23].

In this paper, the mechanism of the effect of the environmental temperature on the sensitivity of an airflow inclinometer will be studied. Then, through numerical simulation and calculation, the equation of the relationship between the inclinometer sensitivity and the variation of the surrounding temperature on the sensing element will be obtained. An optimization model based on GA theory has been presented to obtain a reasonable airflow inclinometer PCB layout so that the sensitivity value of the device achieves a maximum value. Then, using the finite element software ANSYS to simulate and verify the final optimal results, simulation results show that the sensitivity value of the inclinometer is improved by more than 50% with the optimal PCB layout.

## 2. Models and Simulations

Numerical simulations are performed for the temperature field within the chamber under different ambient temperatures by using the finite element software ANSYS-FLOTRAN CFD. The PCB thermal layout optimization model based on GA is established under the MATLAB environment. Simulations for PCB thermal layouts before and after optimization are performed by using the ANSYS thermal analysis tool.

### 2.1. Simulation of the Influence of Ambient Temperature on the Sensitivity of an Airflow Inclinometer

#### 2.1.1. Sensitivity Analysis of an Airflow Inclinometer

Figure 1c is a detection circuit within the airflow inclinometer’s sensing element. *R*_1_ and *R*_2_ are two thermistors, *R* is a precision resistor. The output of the bridge circuit can be expressed as:

(1) $$V_{O}=V_{B}\;\frac{R_{1}-R_{2}}{2(R_{1}+R_{2})}=V_{B}\;\frac{R_{1}-R_{2}}{2r}(\text{unit}:\text{ V})$$

In this formula, *V_B_* is the electrical supply voltage of the bridge circuit, *V_O_* is the output voltage of the bridge circuit, (*R*_1_ + *R*_2_) can be reduced to *r*. When the sensor tilts, it’s certain that one of the thermistors’ values must increase, while the other one will reduce, and it can be considered that the change of absolute values between the two thermistors are approximately equal. This means (*R*_1_ + *R*_2_) remains unchanged, so (*R*_1_ − *R*_2_) determines the output voltage of the bridge circuit when the tilt angle of inclinometer is certain.

Since *R*_1_ − *R*_2_*= α*_1_*R*_0_(*T_a_* − *T_b_*), where *T_a_* and *T_b_* represent the temperature at location of *a* and *b* respectively, *R*_0_ is a thermistor value under condition that the reference temperature is zero degrees Celsius. In general, *α*_1_ = 0.003926 (unit: K^−1^). Equation (1) can be simplified as follows:

(2) $$V_{O}=\frac{V_{B}\alpha_{1}}{2\text{r}}R_{0}(T_{a}-T_{b})(unit:V)$$

where, according to the definitioin: *r* = *R*_0_ when the reference temperature is zero degrees Celsius. According to the actual application, we set *V_B_* = 1.4 V according to the actual parameter, and assuming (*T_a_* − *T_b_*) *=* *ΔT*, the relationship between the output voltage of the bridge circuit and the temperature difference of the two thermistors is as follows:

(3) $$V_{O}=2.7482\times 10^{-3}\times \text{Δ}T(unit:V)$$

According to the Equation (3), the output voltage (*V_O_*) of the bridge circuit is decided by the temperature difference (ΔT) between the two thermistors. According to the definition, the sensitivity of a micro-mechanical airflow inclinometer can be expressed as $V_{O}/\text{Δ}\theta$, *V_O_* is the output voltage of the bridge circuit when the angle of the inclinometer changes by Δθ. Then $V_{O}=2.7482\times 10^{-3}\times \text{Δ}T$ is substituted into $V_{O}/\text{Δ}\theta$. The relationship between the the two thermistors’ temperature difference (ΔT) and sensitivity of the airflow inclinometer is obtained as follows:

(4) $$K=\frac{V_{O}}{\text{Δ}\theta }=\frac{2.7482\times 10^{-3}\times \text{Δ}T}{\text{Δ}\theta }(\text{unit}:\text{ V}{/}^{\text{o}})$$

Here we define that K stands for the sensitivity of airflow inclinometer. From Equation (4), it is found that when the tilt angle of the inclinometer changes, the sensitivity is determined by the changes of the angle of the inclinometer and the temperature difference between the two thermistors. Because Δθ is determined by the posture of the inclinometer, it is not affected by other factors, therefore, it can be concluded that the sensor’s sensitivity and temperature difference (ΔT) between the two thermistors have a one to one relationship. The temperature difference (ΔT) between the two thermistors of detection circuit is thus relevant to the ambient temperature of the sensing element.

#### 2.1.2. Simulation Model for the Sensing Element

**Figure 2:**
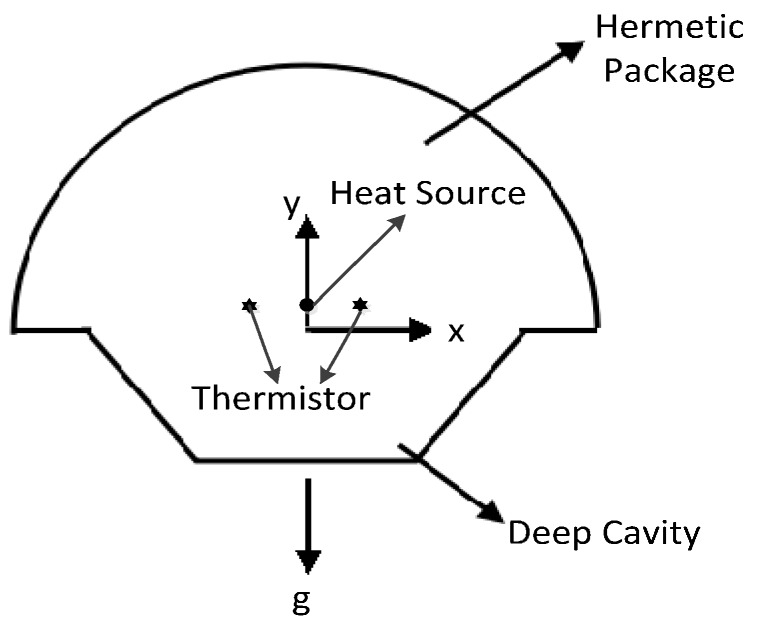
2D structure of the airflow inclinometer’s sensing element.

Since the sensing element for the airflow inclinometer is completely symmetrical along the sensitive axis, the diameter of the resistive heater is far smaller than the diameter of the chamber of the sensing element, so for modeling and calculation efficiency, the chamber of the sensing element can be simplified as a 2D structure, and the resistive heater (the so-called heat source) can be simplified as a central heating point, as shown in Figure 2.

The gas flow area is a 2D semicircular cavity with a inverted trapezoid cavity on its bottom. The sealed chamber is 2.5 mm in diameter, and has two separated points of thermistors, which are 0.83 mm away from each other. The heat source used to generate heat lies in an intermediate position between the two thermistors that are sensitive to temperature, and the outer shell of the chamber maintains cooling. The outer boundary of the hemispherical cavity is set to have the thermophysical properties of stainless steel; while the thermophysical properties of the outer boundary of the inverted trapezoid deep cavity are set as those of silicon.

#### 2.1.3. Simulation of the Temperature Field within the Chamber of the Sensing Element

The aim of the simulation of the temperature field is to study the mechanism of how the airflow inclinometer’s sensitivity is affected by the environmental temperature, as well as present the relationship between the sensitivity of the airflow inclinometer and the ambient temperature. In computations, the thermal properties of the fluid within the chamber are assumed as air-silicon. Assume that the fluid is a Newtonian fluid with constant density, and the flow field is turbulent and incompressible. The turbulent model is adopted for flow field model. The Streamline Upwind Petrov/Galerkin (SUPG) method is used as the momentum equation. The temperature of the heat source at the heart of the chamber is set to be 483 K, which is applied to maintain a stable temperature field within the chamber. The chamber is placed at the 20° tilt angle. The boundary conditions for both sensor package and cavity are regarded as isothermal walls. The ambient temperatures of the chamber boundary are set as 273 K, 288 K, 298 K, 308 K, 318 K, 328 K, 338 K, 348 K, 358 K, respectively, in order to compare the changes of the temperature field within the chamber under different ambient temperatures.

### 2.2. GA Optimization for the PCB Thermal Layout

#### 2.2.1. Genetic Algorithm

The genetic algorithm is an adaptive, global search algorithm. It is based on genetic and evolutionary theory. The genetic algorithm has many advantages, such as adaptivity, artificial intelligence, and strong robustness. It is not the goal of optimizing the mathematical model itself but the code of mathematical model. The parallel operation of genetic algorithm is also another important feature.

#### 2.2.2. Algorithm Processes

**Figure 3:**
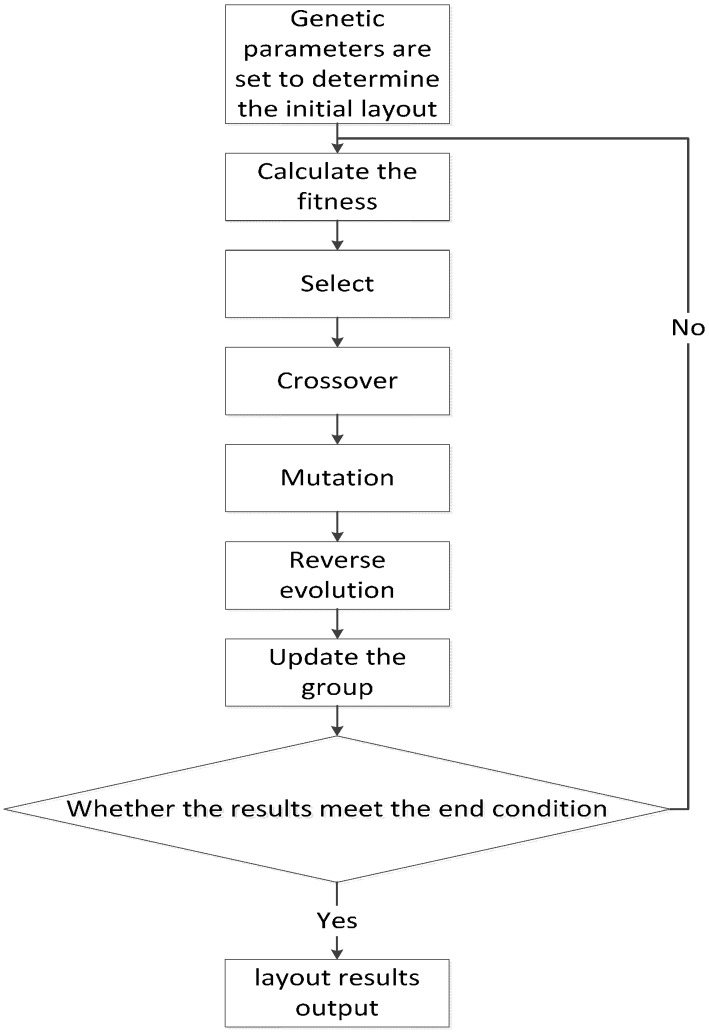
Flow chart of GA.

GA contains processes of selection, crossover, mutation and reverse of the evolution, the flow chart is shown in Figure 3.

#### 2.2.3. GA Calculation Model of the PCB Thermal Layout

To improve the device sensitivity, the sensing element on the inclinometer PCB must be placed at the location which can achieve maximum device sensitivity. From the previous studies we know that the sensing element lies in the lowest temperature areas on the PCB, which is the optimal PCB layout for an inclinometer to achieve the maximum sensitivity. The goal of this study is to reduce the external temperature of the sensing element as much as possible and to achieve the largest sensitivity of the airflow inclinometer by optimizing the components’ placement on the PCB.

One issue of this paper is how to optimize the arrangement of the electronic component, because the different power and kinds of electronic components located at different positions on the PCB, will form a new PCB layout array. All the possible layouts of electronic component are the possible combined state sets, so that we have a system of 10 varied electronic components, whose possible layouts on the PCB number over 10! = 3,628,800 kinds in total. If using conventional methods, searching for an optimal solution in such a large solution space is almost impossible, not to mention the calculation time required when it comes to a layout for a large number of electronic components. The GA has been developed in recent years as a global optimization algorithm, which is particularly applicable for large-scale combinatorial optimization problems.

**Figure 4:**
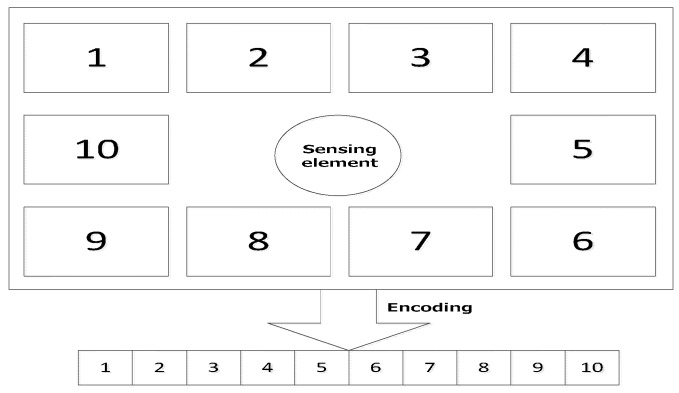
The simplified model for layout optimization and the way of encoding.

In this study, the layout problem of the electronic components on the PCB can be regarded as a plane layout optimization based on the real case. The ultimate goal for the application of GA is to obtain the optimal layout for the electronic components (the global optimal solution of their positional distribution) under certain thermal conditions, and reduce the surface temperature of the sensing element as much as possible, thereby improving the sensitivity of the sensor accordingly. Following these guidelines, the optimal layout of the electronic components on the PCB can be obtained through establishing an optimization model. In order to simplify the calculations, we assume that the electronic components with same material and shape on the PCB have different electric power, so a simplified layout model which consists of the 10 components on the PCB can be established. As it can be seen from Figure 4, the digital numbers correspond to the components’ power and position; for instance component 1 located in the position 1 represents a minimum power component, while e component 10 located at position 10 stands for a maximum power component.

#### 2.2.4. The Setting of Control Parameters

(1) Population size n. Population size affects the execution efficiency of the GA and the final results of genetic optimization. When the population size n is too small, the optimized performance of a GA is generally not very good, and the use of a large-scale population GA can reduce the possibility that the final solution could reach a local optimal solution, but the larger population means higher calculation complexity. Generally, selecting the population size n from 10 to 160 to verify the final results of the corresponding genetic optimization and the operation efficiency of GA in this paper, it is seen through verification that selecting n = 100 is appropriate, and this population can obtain relatively satisfactory optimization results and take a reasonable time for optimization, so for specific problems, we should choose the particular population size in order to search for the best results on the condition that the complexity of calculations is minimized, while keeping a balance between obtaining the optimal results and reducing the optimization time.
(2) Crossover probability P_C_. Crossover is a genetic operator used to vary the programming of a chromosome from one generation to the next. The crossover probability PC controls the frequency of crossover. A larger crossover probability can enhance the probability that the GA opens up new search areas, but if it is too large, will be likely destroy excellent chromosomes. If the value of P_C_ is relatively low, it could affect the search speed of the GA. In general the P_C_ values range from 0.25 to 1.00. In this paper, we compared and tested the crossover probability values 0.8, 0.85, 0.9, 0.95, 0.98, 1.00 in the GA. By means of this process, the results show that selecting P_C_ = 0.9 can make the greatest improvement for the search capabilities of GA, as well as ensuring that the the probability of the destruction of outstanding chromosomes is not large.
(3) Mutation probability P_M_. Variabilities are complementary search operations in GA, whose main purpose is to maintain the diversity of populations’ solutions. Generally, low frequency mutations aim to prevent the possible loss of a single and important gene in the population, the high frequency mutations will enable heredity tends to be a purely random search. Usually the mutation probability is about 0.05, we decided to select 0.05 as P_M_ in this paper too.

#### 2.2.5. Selection of the Fitness Function

In order to meet the demands of the development of miniaturization of airflow inclinometers, the number of components on the corresponding PCBs are increasing, and the placement of these components on the PCB is becoming more and more compact. For these reasons. The power density near the sensing element has increased gradually. When the positions of the electronic components change on the PCB, the surface temperature of the sensing element will change accordingly. The whole PCB layout can be simplified as a 2D layout. The objective of layout optimization is to reduce the surface temperature of the sensing element as low as possible by means of optimization of the thermal layout.

In this paper, applying the temperature equation of the thermal superposition model proposed by Lall *et al.* [24], which is regarded as a the fitness function of the GA, the basic principle is: when it comes to calculating the surface temperature of the sensing element in the inclinometer, the heat generated by self-heating should be considered, in addition, the heat generated by the other components contributing to the sensor should be combined too. Since the calculation of the thermal superposition model involves a Bessel function, in order to reduce computational complexity, a simplified equation is applied to calculate the temperature of the components’ surfaces. The simplified equation of the thermal contribution made by the element electronic components themselves is as follows:

(5) $$T_{io}=15.5\times {\left(1+6.31\times {\left(A_{i}/A_{\max}\right)}^{-2.87}\right)}^{-0.5}(unit:K)$$

where *A_i_* stands for the electric power of electronic component *i*. We use the corresponding digital codes to represent the corresponding 10 components respectively; for the case of 10 components, *A*_max_ stands for the maximum power among the 10 electronic components.

The evaluation equation of the temperature contribution from one component to another one at different distance can be expressed as:

(6) $$T_{ji}=0.0915\times A_{j}/A_{\max}\times {\left(1+3.82\times {\left(D_{ji}/R_{i}\right)}^{-2.36}\right)}^{1.5}(unit:K)$$

where *D_ji_* is the distance between electronic component *j* and electronic component *i*, *R_i_* is the radius of the electronic component, the other variables are the same as in Equation (5). *T_ji_* is the thermal contribution made by electronic component *j* to electronic component *i*, which is inversely proportional to the distance between the two electronic components. This indicates that the further the distance, the less the thermal contribution will be.

Because the GA model only needs to consider the thermal contributions made by different layouts of the electronic components with respect to the sensing element, and the thermal contribution made by the sensing element itself depends on its own power, which is not relevant to the layout of electronic components, so as a result the power consumption of the sensing element itself can be ignored.

The total thermal contributions to the sensing element are calculated as follows:

(7) $$T_{i}=\sum_{j=1}^{10}T_{ji}$$

Among them, *T_ji_* is a thermal contribution value made by electronic component *j* to the sensing element.

(8) $$Fitness=\frac{1}{T_{i}}$$

In the GA field, the fitness function is applied to evaluate the excellence of the final solution, according to the expression showing the fitness function value is the reciprocal of *T_i_*, which means that an individual’s fitness function value is inversely proportional to *T_i_*. In the process of solving the problem, we use the sum of temperature contribution values (*T_i_*) to the sensing element to measure the fitness values, and the greater the sum of temperature contribution values is, the smaller the fitness values are. The fitness values are used to evaluate whether a layout combination solution is optimal.

### 2.3. PCB Thermal Layout Simulations

#### 2.3.1. Selection of the Thermal Analysis Model

According to [25,26], the standard thickness of a PCB is about 1.6 mm, so compared with the length and width of the PCB, its thickness is relatively thin. As a result we can reach the conclusion that the simulation results of a 2D PCB are basically in line with the actual experimental results. On the other hand, 3D simulation models are more complex, and they need to spend more time and energy on modeling and require more specific detailed information for structures and materials. The number of grid related to this falls into a multiple of geometric growth, so the calculation speed will slow down. Although a 3D model may obtain more detailed results, however the 2D simulation can be fast and intuitive, and are able to get an approximate distribution of the temperature field quickly. Because the purpose of the simulations is to obtain the approximate temperature distribution on the PCB, it is thus appropriate to use a 2D model for the simulation in this case of a relatively thin PCB, which will reduce the study workload and avoid the complexity of modeling, and meanwhile provide an approximately accurate temperature distribution on the PCB with the allowable error range.

In the preceding process, the units of available selection for the 2D entity which is solely for the thermal analysis are five species in total, the PLANE75 unit and the PLANE78 unit both belong to the axis of symmetry structure of meshing in ANSYS, which is not appropriate here. The PLANE35 unit and the PLANE77 unit are a three-node triangular element and eight-node quadrilateral element respectively, commonly used in almost shapeless models, so here we choose the PLANE55 four-node quadrilateral element, because it is easy to mesh and solve in the following stage.

#### 2.3.2. Parameter Setting for the Thermal Analysis Model

A PCB board is generally made of a combination of an insulator (such as the material FR4) and copper through a heating and pressurization process. The role of copper is as the conductor of electricity and heat. The thermal conductivity of pure FR4 is 0.35 W/m·K. Thermal conductivity of copper is 388 W/m·K, thus the content and distribution of copper are important factors affecting the overall thermal conductivity. Copper generally exists in the form of copper wire, pads, vias and so on, and their distribution on the PCB is very complex in general, almost impossible for detailed modeling. The thickness of copper has been standardized, and it could be accurately measured, but for copper coverage rates, if we are going to measure and calculate them one by one, it will be very tedious. Thus in order to achieve a balance between calculation accuracy and calculation simplification, an anisotropic plate having a uniform conductivity can be used to simulate the actual PCB according to the thickness of copper and the copper coverage rate on the PCB.

When the electronic components work, they will emit the heat outside because of current conveyance, so in order to verify the availability and accuracy of the optimization program which has been written, here take a numerical example for analysis. The heat generation rate of the 10 components is as follows: 1300 kw/m^2^, 1300 × 2 kw/m^2^, 1300 × 3 kw/m^2^, 1300 × 4 kw/m^2^, 1300 × 5 kw/m^2^, 1300 × 6 kw/m^2^, 1300 × 7 kw/m^2^, 1300 × 8 kw/m^2^, 1300 × 9 kw/m^2^, 1300 × 10 kw/m^2^ respectively, which correspond to each numbered component (from 1–10), and the heat generation rate of the sensing element is set as 1800 kw/m^2^. The thermal conductivities of PCB, the stainless steel packaging of the sensing element and the plastic packaging of the components are 3.8 W/m·K, 20 W/m·K and 0.18 W/m·K, respectively. We set the ambient air temperature of the PCB surface as 25 K, and the thermal coefficient of air convection conversion is 50 W/m^2^·K. The area of the PCB, component and the diameter of the sensing element are set as 28.75 mm × 13.75 mm, 5 mm × 2.5 mm and 2.5 mm, respectively.

#### 2.3.3. Composition of the Thermal Analysis Model

**Figure 5:**
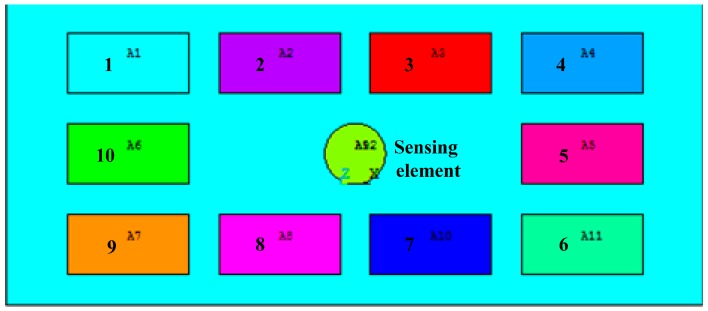
Diagram of simplified PCB thermal model (the digit stands for the position number of the component).

In the analysis and calculation process, the thermal analysis model is seen as three parts: the PCB, the 10 electronic components, and the inclinometer’s sensing element. As shown in Figure 5, the 10 electronic components and sensing element can be regarded as 11 heating blocks.

## 3. Results, Simulation and Discussion

### 3.1. Study Results for the Ambient Temperature Effect on the Sensitivity of the Airflow Inclinometer

#### 3.1.1. Results for the Temperature Field within the Sensing Element Chamber

**Figure 6:**
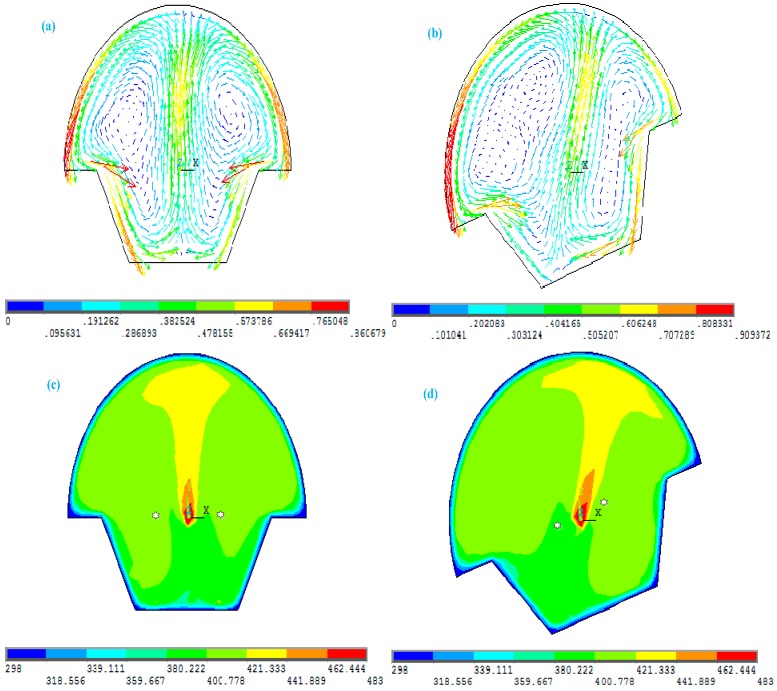
Simulation results for the velocity vector of natural gas convection: (**a**) the direction of gas convection in the horizontal state; (**b**) the direction of gas convection in a tilting state; (**c**) the temperature fields within the chamber in the horizontal state; (**d**) the temperature fields within the chamber in the tilting state (the ambient temperature is set as 298 K).

When the sensor’s sensing element is parallel to the horizontal plane, the hot air generated by a heat source moves vertically upward in the sealed chamber, as shown in Figure 6a, and the temperature fields at the location of the two relatively parallel thermistors are as shown in Figure 6c where the star symbols represent the positions of the two thermistors. The temperature difference between the two is zero. When the sensing element inclines in any direction, the natural direction of gas convection is always opposite to gravity due to effect of natural buoyancy, as shown in Figure 6b. It is discovered that the natural convection of gas has the pendulum characteristics within a closed chamber. The temperature field generated by the heat source remains in the original state, but the opposite positions of the two thermistors have changed, which means changing the relative temperature gradient, and in this case the thermistors are no longer in the same isothermal line, thus the temperature fields at the location of the two thermistors have changed as shown in Figure 6d. There is a certain temperature difference between them, which creates a certain output voltage proportional to the temperature difference (ΔT).

**Figure 7:**
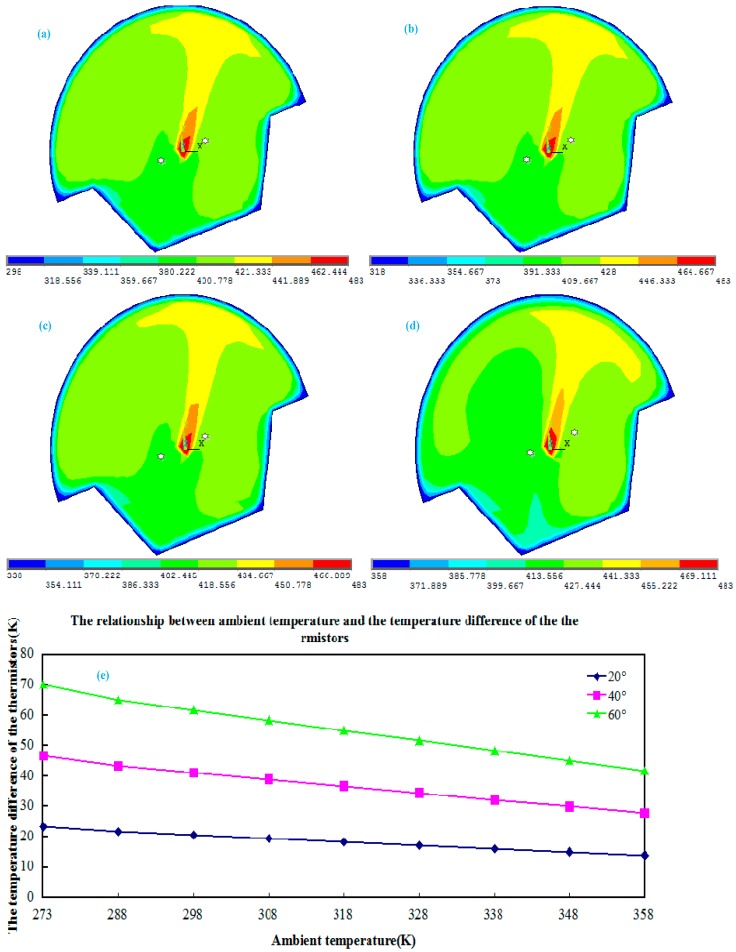
The simulation diagrams of temperature field within the chamber tilted a 20° angle when the ambient temperature is: (**a**) 298 K; (**b**) 318 K; (**c**) 338 K; (**d**) 358 K; (**e**) the line chart for the relationship between ambient temperature and the temperature difference of the thermistors.

In order to compare the two thermistors’ temperature difference for the different changes of ambient temperature, we use the previous simulation methods to calculate the temperature field within the airflow inclinometer’s sensing element for the tilt angle is 20°. Figure 7 shows the diagrams of the simulation result for the temperature field within the chamber tilted at a 20° angle for the different ambient temperatures.

As we can see from the simulation diagrams for the temperature field within the chamber from Figure 7a–d, when the chamber inclines, the temperatures in the position of the two thermistors change accordingly due to the change of ambient temperature, while the line chart in Figure 7e indicates that after the ambient temperature increase the temperature difference of the thermistors decreases. On the contrary, the temperature difference of the two thermistor increases with a decrease of the ambient temperature.

#### 3.1.2. Calculation of the Sensitivity Value

The change of ambient temperature leads to a redistribution of the temperature field near the thermistors within the chamber. When the results of Figure 7e are substituted into Equation (4) then the sensitivity calculation values for the sensor could be obtained as shown in Table 1.

**Table 1:** Calculation of the sensitivity value for the sensor (the temperature of the heat source within the chamber is set as 483 K).

Ambient Temperature	Sensitivity Value		
	20°	40°	60°
273 K	3.206 mV/°	3.206 mV/°	3.206 mV/°
288 K	2.977 mV/°	2.977 mV/°	2.977 mV/°
298 K	2.825 mV/°	2.825 mV/°	2.825 mV/°
308 K	2.672 mV/°	2.672 mV/°	2.672 mV/°
318 K	2.519 mV/°	2.519 mV/°	2.519 mV/°
328 K	2.366 mV/°	2.366 mV/°	2.366 mV/°
338 K	2.214 mV/°	2.214 mV/°	2.214 mV/°
348 K	2.061 mV/°	2.061 mV/°	2.061 mV/°
358 K	1.908 mV/°	1.908 mV/°	1.908 mV/°

Table 1 indicates that the sensitivity of the sensor is affected by the ambient temperature. When the ambient temperature changes, the sensitivity of the sensor changes accordingly. In addition, it shows the tilting position of the sensor is irrelevant to the sensor sensitivity.

**Figure 8:**
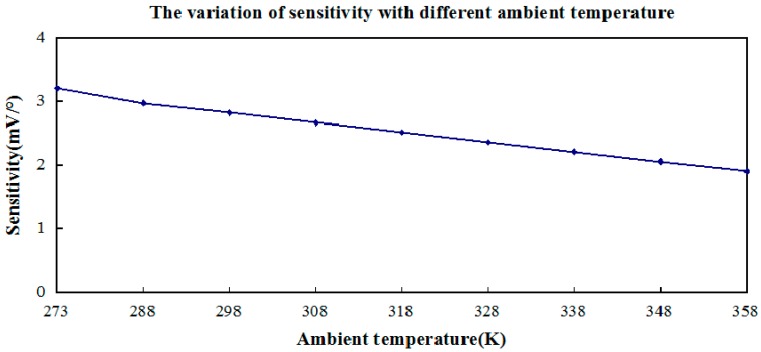
The variation of sensitivity with different ambient temperatures.

It can be seen in Figure 8 that increasing the ambient temperature can simultaneously reduce the sensitivity of the inclinometer. Based on the linear relation between ambient temperature and sensitivity of the inclinometer from the Figure 8, it easy to get the approximate relation formula as follows:

(9) $$S=\text{-}0.0153T_{A}+3.21(unit:mV{/}^{o})$$

where T_A_ represents the ambient temperature, S as sensitivity of the inclinometer.

### 3.2. PCB Thermal Layout Optimization Model Results

**Figure 9:**
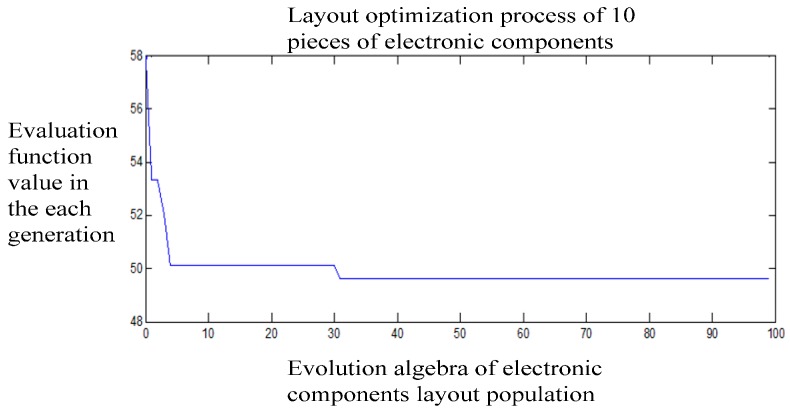
The dynamic process of electronic component layout optimization.

**Figure 10:**
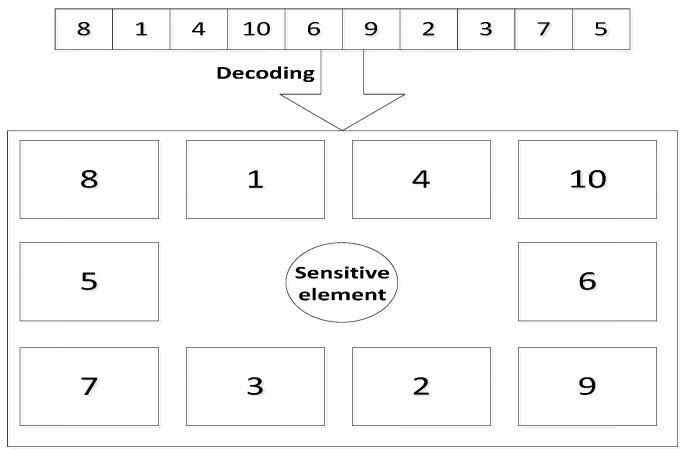
The optimal layout result and decoding of the 10 electronic components.

The optimization model of the layout of the 10 electronic components circuit board through 100 generations of genetic operations makes the evaluation value converge ultimately. This dynamic optimization process as shown in Figure 9, where the abscissa represents the evolution generation of the population of electronic components layouts, the ordinate represents the evaluation function value for each layout generation. In the initial randomly generated population, the corresponding value of the optimal solution is 57.8 K, which stands for the thermal contribution value made by the 10 electronic components to the sensing element. The optimal value converges after about 31 generations in the optimization process, and it can be seen that 49.6 K is the ultimate optimal solution. The optimal layout compared with the initial layout reduces the temperature at the sensing element significantly. The optimal layout of components 8 1 4 10 6 9 2 3 7 5 is also obtained. These code numbers correspond to the 2D layout as shown in Figure 10.

In the optimal layout result seen in Figure 10, the numbers represent the size of the apparent power for each corresponding component, and the PCB layout obtained through optimization places the relatively more powerful electronic components spread away from the sensing element, distributed around the periphery, while the components with small power surround the inclinometer’s sensing element and approach it, which is in agreement with the actual situation.

**Figure 11:**
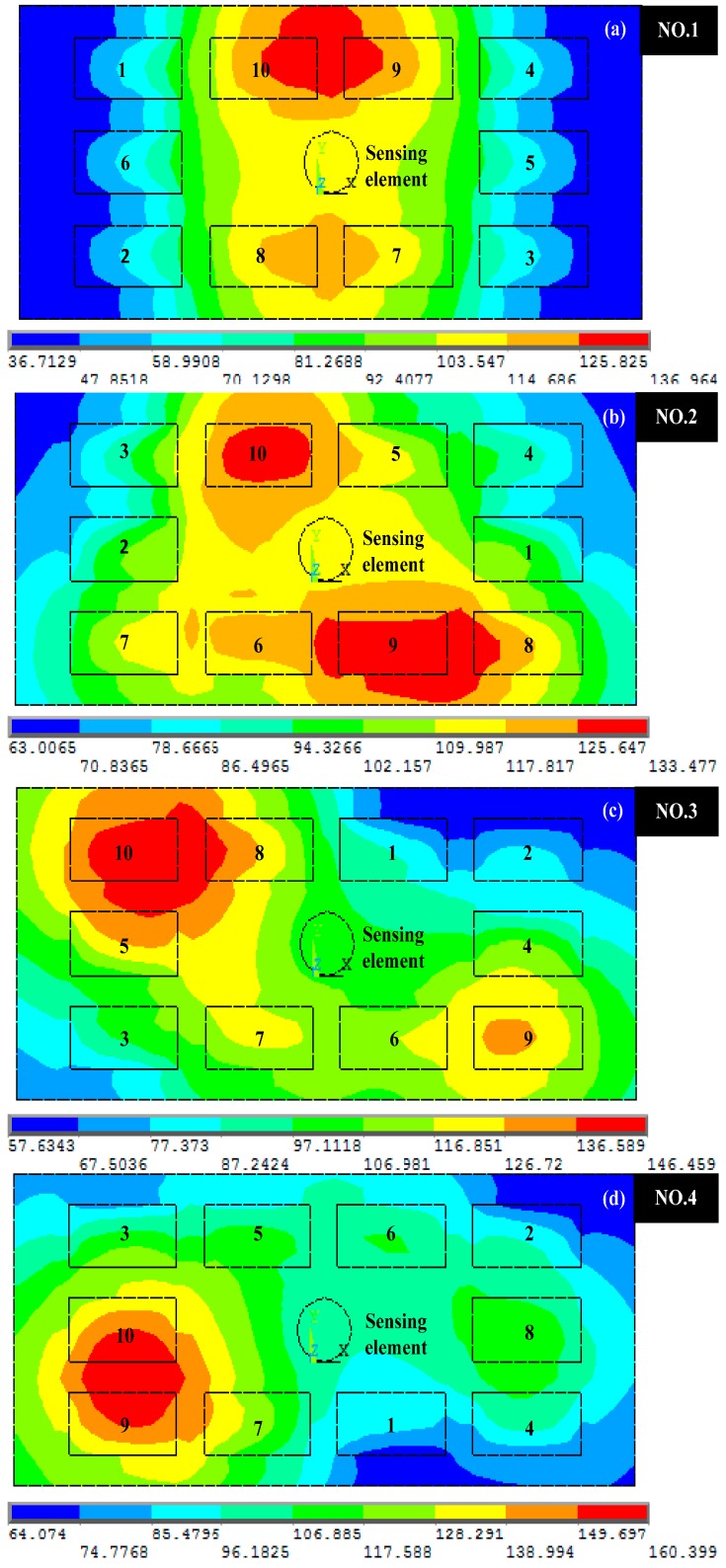
A set of four temperature field distribution diagrams for the random PCB layouts before the layout optimization.

### 3.3. PCB Thermal Layout Simulation Results

#### 3.3.1. PCB Layout Temperature Field Results

Four diagrams for the temperature field distribution of random PCB layouts as shown in Figure 11. As seen in Figure 11, because the high-power electronic components are concentrated around the location of sensing element, this creates a higher temperature around the sensing element than the other parts of the PCB, and this will result in an increase in the surface temperature of the sensing element.

The minimum power components are mostly concentrated on the four edges of the PCB where a relatively low temperature is distributed. In the case of these four PCB layouts it is obvious that the temperatures of the sensing element’s surface are 103.5 K (T_1_ = 103.5 K), 110.0 K (T_2_ = 110.0 K), 97.1 K (T_3_ = 97.1 K), 96.2 K (T_4_ = 96.2 K), respectively.

**Figure 12:**
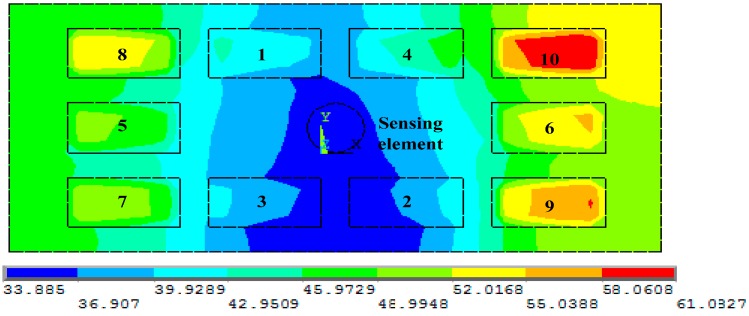
The temperature field distribution after layout optimization.

The temperature field distribution after the GA-based layout optimization is shown in Figure 12. The component arrangement is: 8 1 4 1 0 6 9 2 3 7 5 according to the optimal results in the previous studies. As can be seen from Figure 12, the the high power electronic components are distributed in the vicinity of the four corners of the PCB, while the lower power electronic components are concentrated near the sensing element. For this PCB layout case, the sensing element is situated in the minimum temperature region (T_O_ = 33.9 K) for the entire PCB.

#### 3.3.2. Layout Optimization Before and After Comparison for the Sensor Sensitivity Value

Using Equation (13) to analyze the results, T_1_ = 103.5 K; T_2_ = 110.0 K; T_3_ = 97.1 K; T_4_ = 96.2 K are the temperatures of the sensing element’s surface before layout optimization, and 33.9 K is obtained as the optimal surface temperature of the sensing element after layout optimization. Though Equation (8) the sensitivity values of the sensor obtained before layout optimization are S_1_ = 1.626 mV/°; S_2_ = 1.527 mV/°; S_3_ = 1.724 mV/°; S_4_ = 1.738 mV/°, and after layout optimization the sensitivity value of the sensor becomes S_O_ = 2.691 mV/°. In comparison with the sensitivity value of sensor before layout optimization, the sensor sensitivity values improve by approximately 65.5%; 76.2%; 56.1%; 54.8%, respectively, as shown in Table 2, which means the sensitivity of the airflow inclinometer can be maximized on the optimal PCB layout, proving that the optimal layout results are satisfactory.

**Table 2:** Layout optimization before and after comparison for the sensor sensitivity value.

PCB Number	The Temperature at Sensing Element before Layout Optimization (T_1_; T_2_; T_3_; T_4_)	Sensitivity Values	Improvement Percentage Compared with the Sensitivity Value before Layout Optimization
1	103.5 K	1.626 mV/°	65.5%
2	110.0 K	1.527 mV/°	76.2%
3	97.1 K	1.724 mV/°	56.1%
4	96.2 K	1.738 mV/°	54.8%

The calculation results thus show that the sensor layout obtained though the optimization model based on GA achieves a significant improvement for both the sensitivity and stability of the sensor’s properties.

## 4. Conclusions

Taking into consideration the working mechanism of the airflow inclinometer, the performance of an airflow inclinometer is greatly affected by the ambient temperature changes. To the best of our knowledge, this is the first report of thermal layout optimization for a micro-machined airflow inclinometer’s sensing element using a GA. In order to reduce the influence of the external temperature on the sensitivity of an airflow inclinometer, we present a thermal layout optimization algorithm based on GA and compile the corresponding optimization procedure. The optimal PCB thermal layout was obtained successfully through the GA model. The finite-element method is applied to verify the optimal result through testing a 2D thermal simulation model of the PCB-based airflow inclinometer. The thermal simulation results show that the thermal PCB layout obtained from the GA model makes the sensing element achieve a minimal surface temperature. Calculation results indicate that the sensitivity of the airflow inclinometer achieves a great improvement above 50% after thermal optimization of the PCB layout. As further work, since GA has been demonstrated to have significant applications for optimizing the thermal layout of an airflow inclinometer in this paper, we plan to continue optimizing the PCB layout of the sensors with high efficiency to satisfy various requirements in the industrial field.
